# The Morbid Anatomy of the Liver; Being an Inquiry into the Anatomical Character, Symptoms, and Treatment, of Certain Diseases Which Impair or Destroy the Substance of That Viscus. Order I. Tumors. Part I. On Tubera Circumscripta, and Tubera Diffusa.—Part II. On the Varieties of the Tubera Diffusa

**Published:** 1816-04

**Authors:** 


*The morbid Anatomy of the Liver; being an Inquiry into the anatomical Character, Symptoms, and Treatment, of certain Diseases which impair or destroy the Substance of that Viscus. Order I. Tumors. Part I. On Tubera Circumscripta, and Tubera Diffusa.—Part II. On the Varieties of the Tubera Diffusa.* By J. R. Farre, M.D. large 4to. pp. 52, with four coloured Engravings. London, 1812-1815.

Destined, from its price, to adorn only the libraries of the more opulent of our profession, this work has yet a pre-eminent claim to universal notoriety: and never is the pen of the Reviewer more usefully or laudably exerted than in extending a knowledge of such productions beyond the sphere to which their circulation and influence must otherwise have been limited. The subject, in itself important, is rendered superlatively interesting by the discrimination and talent which its discussion has called forth. Unwilling that our Journal should contain a mutilated record of labours thus valuable, we shall take the liberty of once again giving to our criticism a somewhat wide retrospective operation, so as to include the only two parts of Dr. Farre's publication which have yet appeared, under our present article. By some of our readers this arrangement may be censured, but the majority will applaud it. Many there are in this island, to whose eye, although an indistinct hum of the reputation of Dr. Farre's researches on the liver may have now and then reached them, the "ample page" of the splendid original never has been, or will be, unfolded. They have heard the distant sound of the torrent, but must never quaff the refreshing waters at the brink. Such will be grateful to us for the supply with which we propose to furnish them; scanty though it be, and much of its spirit evaporated, from our imperfect mode of transmission. Nor to these only will our services, probably, be restricted: they whose thirst has heretofore been quenched at the "living stream," may drink of our profered cup with advantage. To resume the sober language from which we have been unwittingly seduced: the present is a work with which every practitioner of medicine should be familiarly acquainted; and even they who have been fortunate enough to peruse the original, can never have the facts and views which it developes too deeply impressed, too frequently recalled to their remembrance.

We are informed by Dr. Farre, in the *Introduction,* that his "work will, at present, be limited to the investigation of Tumors, Scrofulous Affections, and Inflammation of the Liver."

"The structure of the liver is impaired in different modes by these diseases, for tumors are chiefly injurious to it by their unlimited powers of growth or multiplication; but inflammation simply disorganizes the liver by obliterating its structure. Hence the ultimate state of the former is, the utmost degree of enlargement which is compatible with life; of the latter, rather a reduction of bulk, but an increase of solidity. Scrofula only proves destructive to the structure of organs when its tubercles inflame; the disorganizing effect of this disease upon the liver is therefore analogous to that of inflammation."

We have here to consider *Tubera,* the first genus of the Order *Tumores.*

*Definitions.* The term *tumor*, Dr. Farre does not employ in the ordinary acceptation of the word, as "importing simply increased bulk;" but in that sense to which it has been recently restricted by Mr. Abernethy. This will be best understood by transcription from the work itself.

" *Tumores.* Swellings either circumscribed or diffused, generally differing in structure from the natural textures of the affected organs, and increasing in bulk by an inherent growth.—*Tubera.* Tumors of a cellular structure and fungous nature, producing, in general, remarkable elevations from the surfaces of the affected parts.—*Tubera circumscripta.* Tubera determinate in their figure, and limited in their seat chiefly to the liver.—*Tubera diffusa.* Tubera indeterminate in the figure, diffused through the affected organ, or dispersed through many textures of the body."

*Order I. Tumores.* 1. *Tubera.* Under this generical term are included numerous tumors in the liver, connected by evident similarities of character and alliance in kind. It is chosen as expressing the diagnostic sign of such tumors, and indicating their fungous nature.

1. *Tubera circumscripta.* These principally infest the liver.— " *Character.* Their colour inclines to a yellowish-white; they elevate the peritoneal tunic of the liver, and their projecting surfaces, slightly variegated with red vessels, deviate from a regular swell by a peculiar indentation at or near their centres, which are perfectly white and opaque. They vary much in size, which depends on the duration of each tuber; for at its first appearance it is very minute, but during its growth it assumes the character above described, and at its maturity exceeds an inch in diameter. They adhere intimately to the liver, and their figure is well defined. In the interstices of the tubera, the liver is paler and more flabby, its cohesion is weaker than natural, and slight effusions of blood are sometimes found. They commonly remain distinct at the surface of the liver, but internally they ultimately coalesce, and form immense morbid masses, which pervade its substance. The patient often lives until the mass occupies greatest part of the abdomen, and the natural structure of the liver is nearly supplanted. They possess so close a cellular structure, that the section of them, at first view, appears solid and inorganic; but on the edge of the knife by which they have been dissevered, an opaque white fluid, of the consistence of cream, is left, and a fresh portion of this fluid is gathered on it at each time that it is repassed over the surface of the section. Their cellular structure becomes more apparent after long maceration."

*Symptoms.* Pain in the hepatic region, languor, anorexy, cough. Until the liver, from increase of the tubera, descends below the false ribs, correct diagnosis cannot be formed. The chylo-poietic functions are then more impaired. Emaciation begins. The enlargement of the liver, its hardness, and remarkable irregularity of surface, may be felt through the abdominal parietes. At length, distress arises from its enormous bulk; respiration is oppressed; diarrhœa supervenes. Jaundice, or peritoneal effusion, is not essentially connected with the disease; they may exist as accidental complications.

Two cases are circumstantially detailed, in illustration of this species of tuber; we shall select the second, as very clearly elucidating the clinical history and pathologic characters of this intractable malady.—A man, of large stature, aged 39; in his earlier years addicted to drinking; latterly, more temperate; subject to irregular gout, and to severe paroxysms of epigastric pain uncertain in its periods of recurrence, became, towards the close of 1810, affected with cough and uneasy sensations in the region of the stomach and liver. On examination at the end of the ensuing March, the liver projected into the umbilical region. The abdominal parietes, from the navel to the chest, were hard, tumid, and presented a surface irregular from tubera plainly felt beneath the integuments. This enlargement had been, only six weeks before, observed by the patient. Cough and dyspnœa, probably attributable to the great bulk of the liver, were present; pulse natural; appetite defective; bowels somewhat torpid. The circulation of the *vena portarum* and passage of the biliary ducts were unobstructed; for neither peritoneal dropsy nor jaundice had occurred. In April, the man was capable of considerable bodily exertion. Suppression of urine, which came on in May, was relieved by opium. A slight recurrence of the affection in June, yielded to the same remedy. Fluid, in small quantity, was now effused into the peritoneum. By the interposition of this between the peritoneal tunic of the liver and other peritoneal surfaces, the former uneasy sensations were much diminished. Diarrhœa set in. The gibbous margins of both lobes of the progressively-enlarged liver were, at this period, distinguished much below the navel. The chylo-poietic functions were now deranged; tongue red and glossy; appetite gone; pulse frequent; cough and dyspnœa aggravated; expectoration of thick mucus; profuse sweats; emaciation rapid; and loss of muscular power. Death happened on the 5th of July. The only medicinal remedies employed were, rhubarb as an aperient, and opium as a sedative and soporific.

"The body was examined on the following day.—Abdomen. The liver occupied the hypochondriac, epigastric, and umbilical regions. A line drawn across the anterior superior spinous processes of the ilia would have defined its extent, so that, a few convolutions of the small intestines in the hypogastric region excepted, the diseased liver alone appeared after the section of the abdominal parietes had been completed. The liver was removed. Its surfaces were covered with tubera; more than fifty were counted on its concave surface, and on its convex surface there was a still greater number. All these tumours had the same character, a circular margin, elevated, firm, and white, with a depressed and very white centre, resembling a cicatrix. The only deviation from this character was occasioned by a coalescing of two tubera, by which their figure was changed to an irregular oval. Beside these mature tubera, a countless number of them, in their incipient state, appeared in all directions. Sections of the liver exposed the bodies of these tumours, which were of various sizes, and readily distinguished from the natural structure by their yellowish white colour. From their cut surfaces a thick fluid could be scraped. The hepatic artery was enlarged, the vena portæ and venæ hepaticæ were natural; the hepatic duct was dilated, and tinged yellow; the cystic duct was contracted; the gall bladder was elongated, and contained a little bile The mucous coat of the stomach, and of some portions of the intestinal canal, had an erythematous, but no where an ulcerated appearance. The fæces were of a pale yellow colour. The spleen was simply enlarged to four times its natural size. In the pelvis of the left kidney a small calculus rested. The quantity of serum effused into the peritoneum was very inconsiderable."

The thoracic organs were sound; the internal membrane of the aorta of a dark red hue. The morbid state of the liver here described, is illustrated by a singularly beautiful engraving. In one variety of the T. circumscripta, observed by Dr. Farre, the tubera were smaller, and of a firmer consistence.

This affection is identical with the large white tubercle of the liver described by Dr. Baillie, and which that distinguished pathologist infers, from somewhat loose analogy, to be of a scrofulous nature. Dr. Farre entertains an opposite opinion. He regards the T. circumscripta as essentially different in their nature from scrofula, although they sometimes exist in combination. Two cases have fallen within his notice, in which the former malady was complicated with scrofulous lesion of the mesenteric glands. The following are the grounds upon which he dissents from the opinion of Dr. Baillie:

"First, The tubera circumscripta are distinctly allied to the tubera diffusa, which unquestionably fall under the tribe of fungous diseases. Secondly, The tubera circumscripta differ from the tubercula strumosa in their character and termination."

2. *Tubera diffusa.* To the attack of this species, the various textures of the body are as obnoxious as the liver.

" *Character.* These tumors not only pervade the substance of the liver in a distinct or in a confluent form, but also appear at its surface, elevating more or less its peritoneal tunic. They rise from the surface of the liver with a more gradual and uniform swell than the tubera circumscripta, and are, in different subjects, of various figures, sizes, colours, and consistence, often pulpy. No texture seems to escape the ravages of this fungus. It appears indifferently in all the viscera, in the cellular membrane, and even in the bones."

*Symptoms.* Variable, according to the seat of the morbid structure. When the liver is affected, they little differ from those attendant on the T. circumscripta.

The varieties of this disease are numerous. The co-existence of tubera in different textures prevails through all. Dr. Farre selects, for the purpose of description, " that form, which is not only remarkable for its magnitude, but for its greater similitude to the tubera circumscripta."

From the two cases of T. diffusa, recorded by Dr. Farre, we shall transcribe that in which the characters of this species of tuber are most minutely delineated. The history of symptoms is very imperfect. It was communicated to Dr. Farre. The liver and stomach were submitted to his examination. The subject, an adult male, suffered, in the autumn of 1809, from cough and diarrhœa, succeeded by peritoneal effusion and enlarged liver, which descended below the umbilicus. Hydro-thorax came on. Death took place after six months. Mercury, nitric acid, purgatives, blisters, and finally palliatives, had been employed. —Dissection, Liver tuberous and enormously large. Gastric mucous membrane affected with the same disease. Serous effusion into the peritoneum, pericardium, and left pleura; adhesion of the right pleura.

Dr. Farre found the weight of the liver to be more than fifteen pounds.

" This remarkable increase of bulk depended on the growth of tubera, which differed from the tubera circumscripta in the following circumstances. They were less numerous, for on the concave surface of the liver their number did not exceed twelve; they were as minute at their beginning, but at their maturity considerably larger, their dimensions being then rather more than two inches: at no period of their growth were they externally indented, but, on the contrary, they rose from the liver with a gentle and uniform swell, each being either round or oval; their external surfaces had a motley appearance, their white colour mingling with the brown colour of the liver; but a section displayed the appearance of the tubera distinct from the substance of the liver, on which they seemed continually to encroach, and to approximate to each other by waving margins; their texture was coarser, but it yielded a similar whitish fluid. The vessels of the liver were not thickened, neither did the trunks of the artery, vein, or duct, present any thing worthy of remark. The gall bladder and cystic duct were much elongated, and the former contained some bile of a yellow colour. From the mucous coat of the stomach, near the cardia, a cluster of tubera grew, and projected into the cavity; but the disease in this organ was more incipient. did not interrupt the course of the alimentary matters, and, being connected with such extensive hepatic disease, was not suspected."

The morbid characters are finely expressed, pl. ii. fig. 1.

This species of tuber is, in common, more rapidly fatal than the preceding; many organs simultaneously suffering from its ravages. The next case (fourth of Dr. Farre) strikingly illustrates that dispersion through various textures which constitutes the principal feature of the species, and the varying character of its external phænomena. These latter were violent pain of the head, recurring periodically; rapid pulse; white tongue; nausea; cough; succeeded by vomiting, progressively aggravated delirium, convulsion, insensibility, death. There was effusion of fluid into the ventricles of the brain. That organ, the left cervical region, the bronchial glands, the left kidney and liver, were affected by tubera diffusa. The latter viscus exhibited but one tuber, which, as far as an opinion can be formed from relative size, Dr. Farre believes to have been of more recent origin than the tubera of the other morbid organs. The liver is not unfrequently exempt from this disease, even when it exists in other viscera.

There are some remarks, made towards the close of this part by Dr. Farre, which we cannot resist the temptation of transcribing:

"The investigation of disease by anatomy not only improves the diagnostic part of medicine, by connecting as far as it can be done, the sign with the morbid change, but it also improves the therapeutic, by gradually separating curable from incurable disease, or by indicating the stage at which the former is converted into the latter. It is, therefore, one important use of morbid anatomy to point out the boundaries beyond which it is not only unavailing, but injurious, for art to interfere, except to diminish suffering."

Mercury is inadmissible in both forms of the disease which we have been considering; "for by the time that the most careful examination can distinguish them, the progress of the disease has been already so considerable, that the mercurial action tends only to exhaust powers, which art will subsequently in vain attempt to restore." After eulogizing the palliative plan, pursued in the second case of T. circumscripta, as comprehending all that art can safely attempt, or usefully accomplish, in these forlorn maladies, Dr. Farre concludes with the following judicious and memorable sentence :

" This view of the subject is not derogatory ; for the perfection of medicine consists, not in vain attempts to do more than nature permits, but in promptly and effectually applying its healing powers to those diseases which are curable, and in soothing those which are incurable."

( To be concluded in our next. )

---

